# Hierarchical change-point regression models including random effects to estimate empirical critical loads for nitrogen using Bayesian Regression Models (brms) and JAGS

**DOI:** 10.1016/j.mex.2022.101902

**Published:** 2022-10-30

**Authors:** Tobias Roth, Simon Tresch, Enzai Du, Sabine Braun

**Affiliations:** a Zoological Institute, University of Basel, Vesalgasse 1, Basel CH-4051, Switzerland; b Hintermann & Weber, Austrasse 2a, Reinach CH-4153, Switzerland; c Institute for Applied Plant Biology, Benkenstrasse 254a, Witterswil CH-4108, Switzerland; d Faculty of Geographical Science, Beijing Normal University, Benkenstrasse 254a, Beijing 100875, China

**Keywords:** Empirical critical loads, Nitrogen deposition, Change-point regression models, Broken-stick regression models, Piecewise regression models, Segmented regression models, Gradient studies, Foliar N:P ratio in coniferous trees

## Abstract

The concept of critical loads is used in the framework of the Convention on Long-range Transboundary Air Pollution (UNECE) to define thresholds below which no damaging effects on habitats occur based on the latest scientific knowledge. Change-point regression models applied in a Bayesian framework are useful statistical tools to estimate critical empirical loads. While hierarchical study designs are common in ecological research, previous methods to estimate critical loads using change-point regression did not allow to analyse data collected under such a design. This method update provides an implementation of hierarchical data structure by including random effects such as study sites or as in this example tree species within the Bayesian approach of change-point regression models using two different approaches. The example data set is an European wide gradient study of the impact of climate change and air pollution on forest tree health assessed by foliar nutrient status of nitrogen (N) to phosphorus (P) from 10 different conifer tree species originated from 88 forest sites and 9 countries covering 22 years (1995-2017). Both modelling approaches using JAGS and Bayesian Regression Models using ‘Stan’ (brms) resulted in reasonable and similar estimations of the critical empirical load for nitrogen (CL_emp_N) for temperate forests. These methodological examples of using different approaches of Bayesian change-point regression models dealing with random effects could prove useful to infer CL_emp_N for other ecosystems and long-term data sets.

• Hierarchical change-point regression models are suitable for estimating critical empirical loads.
• The Bayesian framework of these models provides the inclusion of the current critical load and various confounding or modifying variables.
• Here we present two ways of implementing hierarchical data sets in Bayesian change-point regression models using JAGS and brms.

## Highlights

• Hierarchical change-point regression models are suitable for estimating critical empirical loads.
• The Bayesian framework of these models provides the inclusion of the current critical load and various confounding or modifying variables.
• Here we present two ways of implementing hierarchical data sets in Bayesian change-point regression models using JAGS and brms.
• The used model data are foliar N:P ratios of 10 different conifer tree species from 88 European forest sites and 9 different countries covering 22 years (1995-2017).

## Graphical abstract

**Figure:**
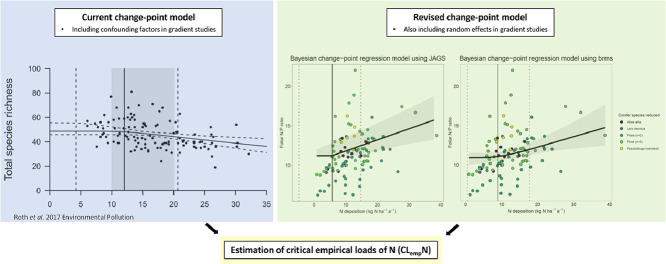

**Specifications table**

**Table:** 

Subject area	Environmental Science
More specific subject area	Forestry research
Method name	Revised Bayesian change-point regression models including random effects using brms and JAGS
Name and reference of original method	Change-point models applied in a Bayesian context by [1]
Resource availability	Model data can be found in [2]. Codes including models and graphics are written in R [3]and given as an RMarkdown [4] output. The MCMC simulations of the change-point modelswere conducted using JAGS, version 4.3.0 [5], executed in R using rjags [6] and in STAN with brms [7].

## Method details

### Bayesian change-point regression model settings

The revised change-point models using either JAGS or brms are based on the current method of using Bayesian change-point models as in the example given by [8]. The Bayesian analysis is based on MCMC methods [9] with a similar setting used by [8] conducted using JAGS (version 4.3.0 [5] and executed in R using *rjags* [6]. Posterior distributions were based on parallel chains (t.n.chains=2) with 100000 iterations each (t.n.iter=100000), discarding the first 50000 values (t.n.burnin=50000) and thinning the remainder by 2 (t.n.thin=2). The two parallel chains were used to assess convergence with Gelman and Rubin’s diagnostics [10] using trace and marginal density plots (Fig. 2) with the R function *plot{coda}* and scale reduction factors with the R function *gelman.diag{coda}* using the R package *coda* [11]. We are presenting the results as the mean (point estimate) and the 2.5% and 97.5% quantiles (95% credible intervals (CI), [12]) of the posterior distribution following [13].

### Model data set

The used data set is a European wide gradient study of the impact of climate change and air pollution (N deposition) on forest tree health assessed by foliar nutrient status N:P of different different conifer species (n=10) from the years 1995-2017 [2]. N deposition are based on the EMEP MSC-W model with a 0.1° spatial resolution [14]. Mean annual temperatures (MAT) are based on the E-OBS climate data set with a 0.25° spatial resolution [2].

### Prior settings

We used the approved critical load for coniferous woodland, which is currently set at 5-15 kg N ha^-1^ a^-1^ [15], to construct an informative prior (CL_emp_N) assuming a normal distribution with the approved critical load as its mean and half the range as its standard deviation (Normal(mean = 10, sd = 5)) and vague priors with mean = 0 and sd = 2 for fixed effects such as the mean annual temperature (MAT) in this example.

### Bayesian change-point regression model using JAGS (BCR_JAGS)

**Fig. 1:**
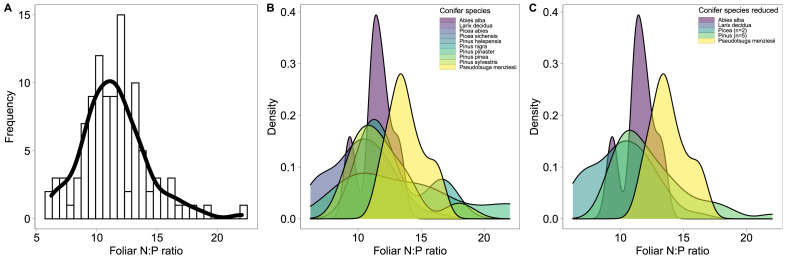
Data distribution of the data set of European foliar N:P ratio. A: Frequency plot of all data including 10 conifer tree species. B: Density distribution grouped by conifer species. C: Density distribution grouped by the different genus types of conifer species, which was used in the final change-point regression model as random effect.

According to the current Bayesian framework of change-point regression models by [1], we used a linear mixed effect model (c.f. data distribution in Fig. 1) with identity-link function. We described the variation of measured foliar N:P ratio (*NP_i_*) between i=1,...,N sampling sites using a Normal distribution with expected foliar N:P ratio $\lambda_{i}$:

$NP_{i}∼Normal\left(\lambda_{i}\right)$ and the expected foliar N:P ratio $\lambda_{i}$ as identity link function expressed as:

$\lambda_{i}=\beta_{0}+\sum_{k=1}^{K}\beta_{k}X_{i,k}+\alpha_{i}$ with $\beta_{0}$ as intercept and $\beta_{k}$ as linear slopes for k=1,...,K confounding variables at sampling site *i* with covariate value $X_{i,k}$ [16]. According to [1] we added $\alpha_{i}$ as the effect of N deposition $N_{i}$ on the expected foliar N:P ratio $\lambda_{i}$:

$\alpha_{i}=\{$$\begin{array}{c} 0\;if\;N_{i}<CL\\ \beta_{N}\left(N_{i}-CL\right)\;if\;N_{i}\geq CL \end{array}$ assuming no effect of N deposition if N deposition $N_{i}$ is below the critical load (Cl) and a linear change ($\beta_{N}$) of $\lambda_{i}$ with increasing N deposition if $N_{i}$ is equal or above the critical load (CL).

The random effect term (lines 20-22 in the JAGS model: JAGS_change_point_model_random_effect.R) was defined as:

$\lambda_{i}=\beta_{0,s}+\sum_{k=1}^{K}\beta_{k}X_{i,k}+\alpha_{i}$

$\beta_{0,s}∼norm\left(\mu sd\right)$ with the intercept $\beta_{0,s}$ that varies between conifer tree species in this example. The differences between the tree species is modelled using a normal distribution, which is the definition of a random effect.

#### BCR_JAGS model diagnostics

**Fig. 2:**
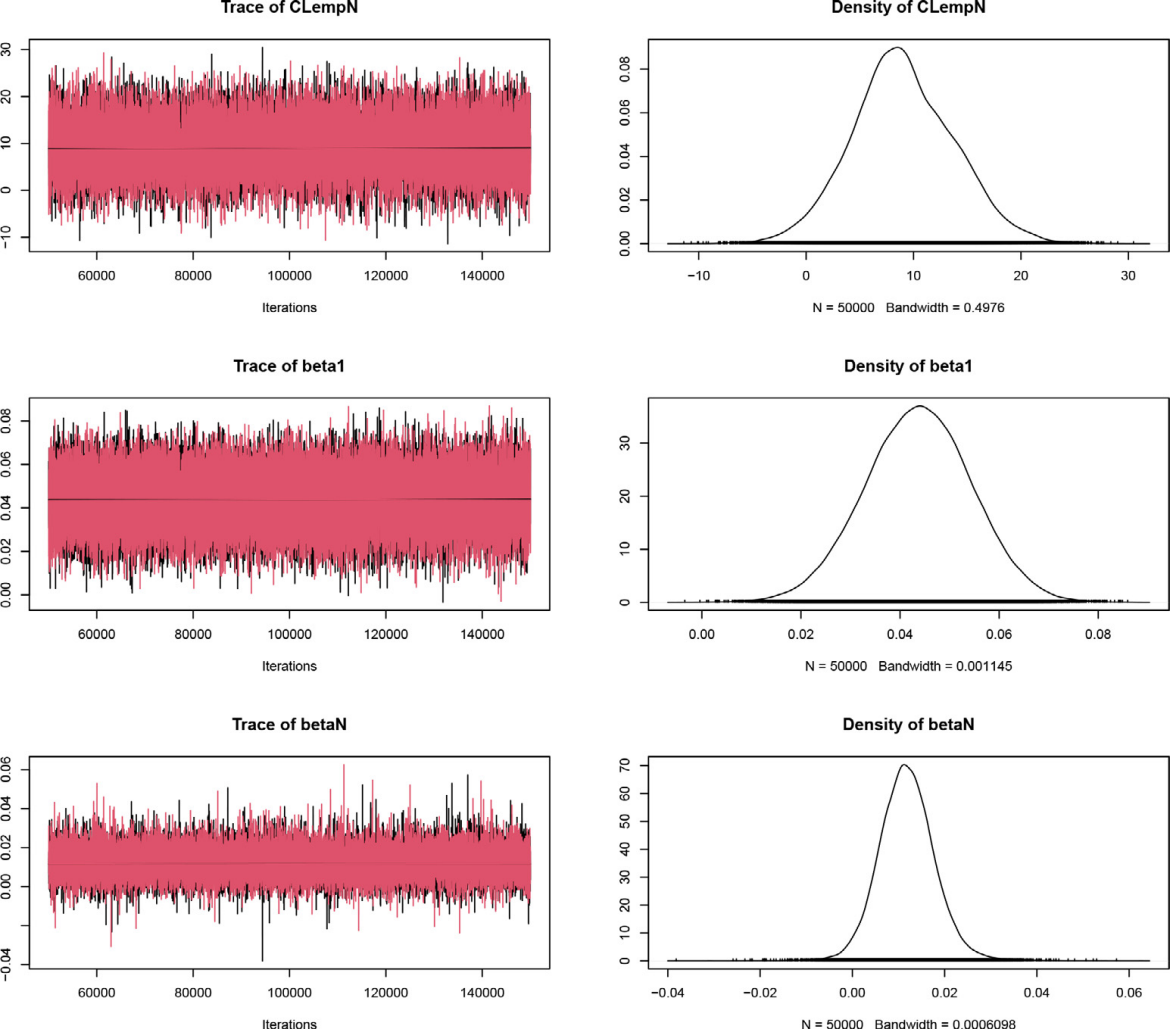
Bayesian change-point regression model convergence diagnostics of the model BCR_JAGS, including the proposed integration of the random effect. Left: Trace plots showing the values of the three model parameters during the iterations of the two chains (black and red). Right: Density plot of the three model parameters, showing the distribution of the values in the chains.

The convergence diagnostics using Gelman and Rubin’s [10] trace and marginal density plots (Fig. 2) showed a good convergence of the two chains. Also the smoothed density plots showed rather balanced histograms of the trace plot values for this model.

#### Estimated change-point using JAGS

**Fig. 3:**
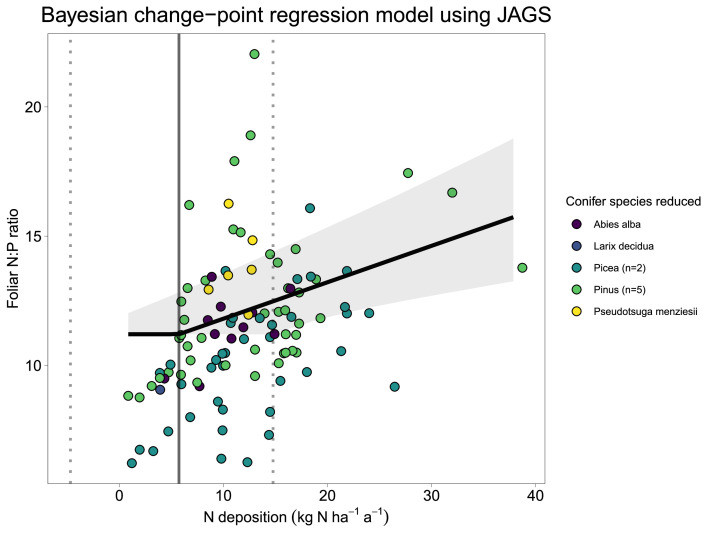
Change-point regression (model BCR_JAGS) of foliar N:P ratios including the fixed effect MAT and tree species as random effect. The grey line represents the estimated critical load (Table 1) of N including the corresponding 95% credible intervals as dotted lines. Points are measurements from [2] and are coloured according to conifer tree species. The black line is the estimated change-point regression including 95% credible intervals.

The effect plot of the estimated change-point including the random effect of conifer tree species is shown in Fig. 3. The change-point, using the underlying data on foliar N:P ratio and the Bayesian change-point regression model BCR_JAGS, is 5.7±4.8 (Tab.1).

### Bayesian change-point regression model using brms (BCR_brms)

With the package “brms” [7] we fitted a Bayesian non-linear multivariate multilevel model with random intercept ($\beta_{0}$) and different CL_emp_N between the tree species:

$\begin{array}{c} brmsformula(\\ dependvar∼\beta_{0}+\beta_{1}*fixeff+\\ step(Ndep-CLempN)*betaN*(Ndep-CLempN),\\ CLempN\;1+(1|random),\\ beta0\;1+(1|random),\\ beta1+betaN∼1,\\ nl=TRUE,family=gaussian) \end{array}$

With the same priors and model settings as before in the model BCR_JAGS (chains = 2, iter=100000, thin = 2, warmup = 50000, cores = 4).

#### BCR_brms model diagnostics

**Fig. 4:**
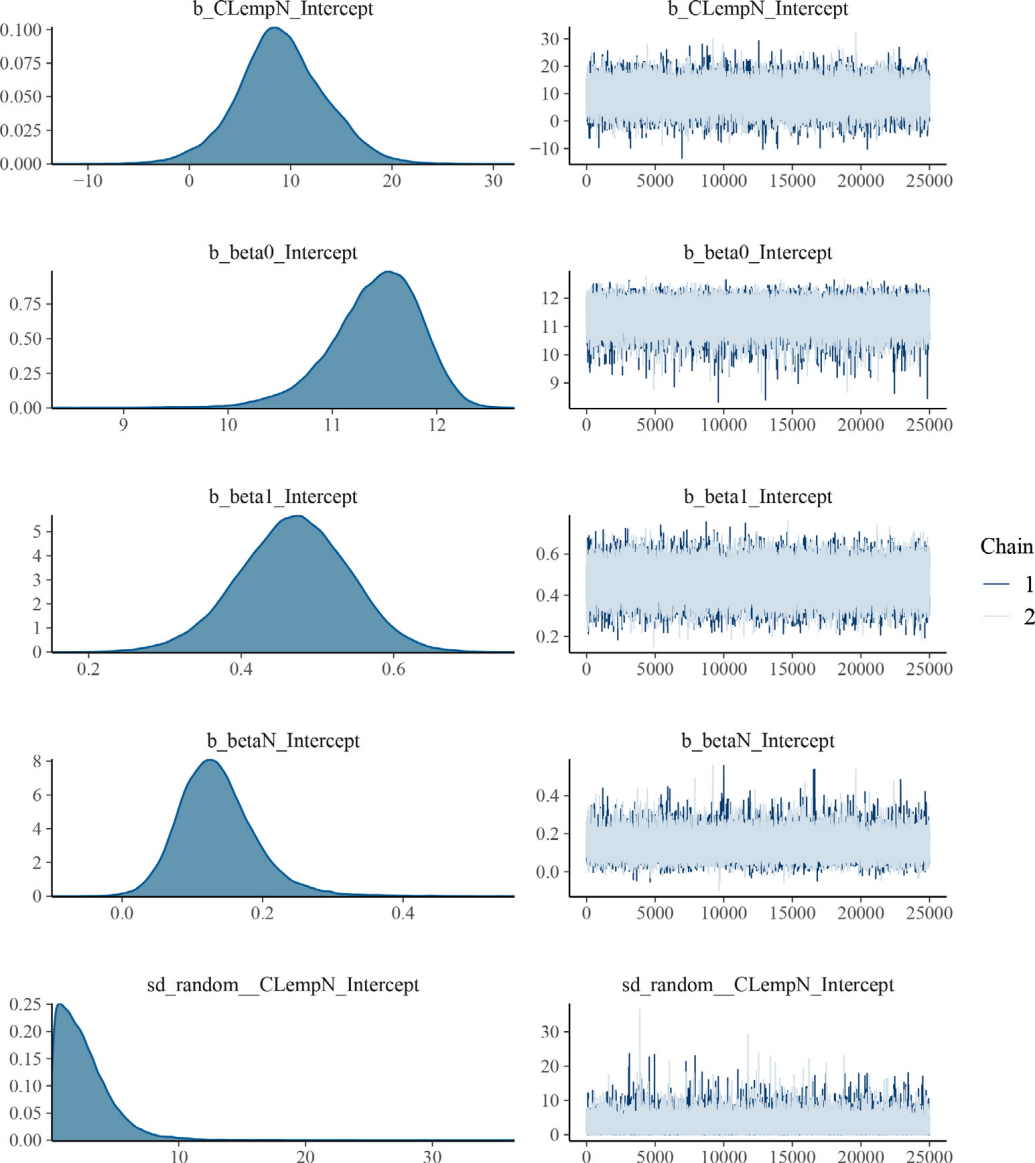
Bayesian change-point regression model convergence diagnostics of the model BCR_brms. Right: Trace plots showing the two chains. Left: Density plot of the posterior distributions.

The MCMC diagnostics showed a good convergence of the two chains. The posterior distributions are centred around one peak value (Fig. 4).

#### Estimated change-point using brms

**Fig. 5:**
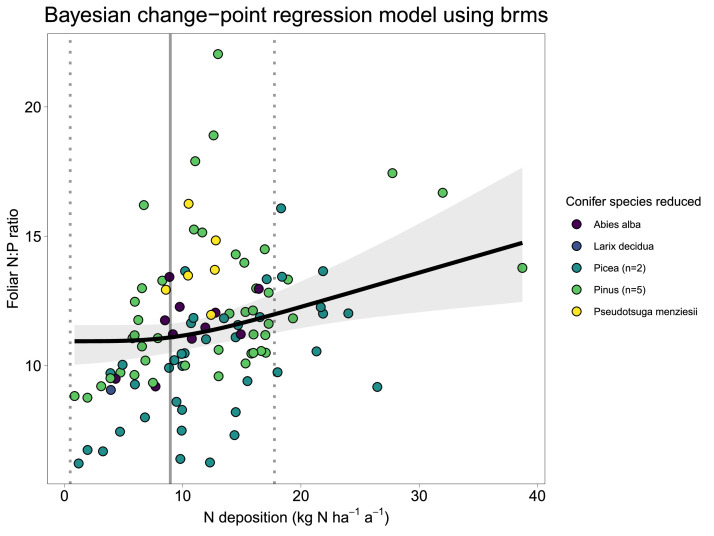
Change-point regression (model BCR_brms) of foliar N:P ratios including the confounding factor mean annual temperature (MAT) and tree species as random effect. The grey line represents the estimated critical load (Tab. 1) of N including the corresponding 95% credible intervals as dotted lines. Points are measurements from [2] and are coloured according to conifer tree species. The black line is the estimated change-point regression including 95% credible intervals.

The estimated change-point using the model BCR_JAGS is 9.0±4.3 (Table 1) with an estimated Bayesian R^2^ = 0.42 [17]. The effect plot of the change-point is shown in Fig. 5.

**Table 1:** Estimated critical empirical loads of N with the change-point regression model BCR_JAGS and BCR_brms. Estimated model parameters are given as the mean of the Bayesian posterior distribution and the 95% credible intervals (CI).

Estimated CL_emp_N (kg ha^-1^a^-1^)	Mean	SD	CI 2.5%	CI 97.5%
BCR_JAGS	5.71	4.81	-4.69	14.81
BCR_brms	9.06	4.31	0.49	17.76

## Method validation

The two presented ways of modelling change-point regressions including random effects, with JAGS (BCR_JAGS) and with the brm function from the brms [7] package (BCR_brms), resulted in similar estimated CL_emp_N (Table 1), latter being slightly higher with the model (BCR_brms). The estimated CL from the model (BCR_brms) including the non-linearity and the random intercepts of $\beta_{0}$ made the outcome more realistic compared to the underlying data (Fig. 5).

**Fig. 6:**
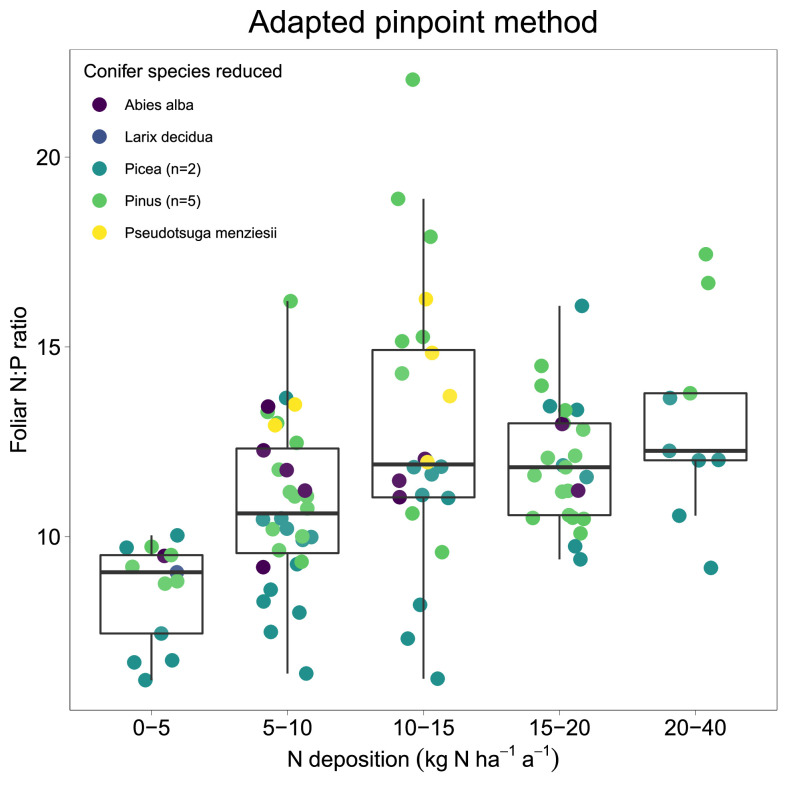
Changes in foliar N:P ratio with increasing N deposition values. The group-wise boxplot comparison (pinpoint method according to [15]) highlights a suggested change in foliar N:P ratio between the first (0-5 kg ha^-1^ a^-1^) and the second group (5-10 kg ha^-1^ a^-1^).

In addition, a simple boxplot comparison (pinpoint method applied by [15]) of different groups of N deposition is suggesting a change in foliar N:P ratio between the first group of 0-5 kg ha^-1^ a^-1^ and the second group of 5-10 kg ha^-1^ a^-1^ (Fig. 6), which verifies the estimated CL_emp_N of both models.

## Additional information

### Introduction to critical empirical loads and change-point regression models

The deposition of atmospheric nitrogen (N) is a major threat to biodiversity and ecosystem functioning globally [18], [19], [20], [21], [22]. The accumulation of N over time is one of the main drivers of changes in species distribution across many habitats, especially for habitats with low management regimes such as natural forests [15]. The impacts of N deposition are known for certain terrestrial habitats, but many still remains uncertain [15]. Critical loads of N has been developed in the framework of the Convention on Long-range Transboundary Air Pollution [15], [23]. These critical loads are defined as thresholds below which damaging effects on specific habitats do not occur based on the latest scientific knowledge [24]. Critical loads of N are often defined based on negative effects on plant diversity [20], as they are often lower compared to critical loads of N from soil processes such as soil acidification or N leaching [25]. Empirical critical loads can be determined with the use of N addition or reduction experiments or in gradient studies covering a gradient of N deposition. Latter is a more holistic approach taking into account the variability of natural habitats. However, gradient studies can also have various limitations for instance they should use a sufficient spatial scale of the air pollutant assessed and they should take into account the most important modifying factors [26]. In addition, an appropriate framework of regression model should be selected, suitable for the selected study design. Bayesian change-point regression models have been shown to overcome some difficulties of large spatial variation found in natural habitats by taking into account confounding factors such as climatic variation or different soil types [23], [27], allowing a more accurate estimation of the critical load [28]. Therefore, change-point regression models applied in a Bayesian framework are useful statistical tools in estimating critical empirical loads [1].

## Declaration of Competing Interest

The authors declare that they have no known competing financial interests or personal relationships that could have appeared to influence the work reported in this paper.

## Appendix A Supplementary materials

Supplementary Data S1 RMarkdown output as .pdf file with all R codes used for this paper. This is open data under the CC BY license http://creativecommons.org/licenses/by/4.0/ Supplementary Data S1

Supplementary Data S2 R Project file. This is open data under the CC BY license http://creativecommons.org/licenses/by/4.0/ Supplementary Data S2

Supplementary Data S3 Raw data as .csv file. This is open data under the CC BY license http://creativecommons.org/licenses/by/4.0/ Supplementary Data S3

Supplementary Data S4 JAGS code file. This is open data under the CC BY license http://creativecommons.org/licenses/by/4.0/ Supplementary Data S4

Supplementary Data S5 Metadata description as .txt file. This is open data under the CC BY license http://creativecommons.org/licenses/by/4.0/ Supplementary Data S5

Supplementary Data S6 RMarkdown codes. This is open data under the CC BY license http://creativecommons.org/licenses/by/4.0/ Supplementary Data S6

## References

[1] Roth T., Kohli L., Rihm B., Meier R., Achermann B. (2017). Using change-point models to estimate empirical critical loads for nitrogen in mountain ecosystems. Environ. Pollut..

[2] Du E., van Doorn M., de Vries W. (2021). Spatially divergent trends of nitrogen versus phosphorus limitation across European forests. Sci. Total Environ..

[3] R Core Team, Team R Development Core, R: A Language and Environment for Statistical Computing, 2021, http://www.r-project.org/.

[4] J.J. Allaire, Y. Xie, J. McPherson, J. Luraschi, K. Ushey, A. Atkins, H. Wickham, J. Cheng, W. Chang, R. Iannone, rmarkdown: Dynamic Documents for R, 2021. https://github.com/rstudio/rmarkdown.

[5] Plummer M. (2003). JAGS: A Program for Analysis of Bayesian Graphical Models using Gibbs Sampling. 3rd Int. Work. Distrib. Stat. Comput. (DSC 2003); Vienna, Austria.

[6] M. Plummer, rjags: Bayesian Graphical Models using MCMC, 2019. https://cran.r-project.org/package=rjags.

[7] Bürkner P.-C. (2017). brms : An R Package for Bayesian Multilevel Models Using Stan. J. Stat. Softw..

[8] Roth T., Sprau P., Naguib M., Amrhein V. (2012). Sexually selected signaling in birds: A case for Bayesian change-point analysis of behavioral routines. Auk.

[9] Link W.A., Cam E., Nichols J.D., Cooch E.G. (2002). Of Bugs and Birds: Markov Chain Monte Carlo for Hierarchical Modeling in Wildlife Research. J. Wildl. Manage..

[10] Brooks S.P., Gelman A. (1998). General Methods for Monitoring Convergence of Iterative Simulations. J. Comput. Graph. Stat..

[11] Plummer M., Best N., Cowles K., Vines K. (2006). CODA: Convergence Diagnosis and Output Analysis for MCMC. R News.

[12] Gelman A., Greenland S. (2019). Are confidence intervals better termed ǣuncertainty intervalsǥ?. BMJ.

[13] Korner-Nievergelt F., Roth T., Von Felten S., Guélat J., Almasi B., Korner-Nievergelt P. (2015).

[14] Fagerli H., Tsyro S., Jonson J.E., Nyíri Á., Simpson D., Wind P., Benedictow A., Klein H., Mu Q., Denby B.R., Wærsted E.G. (2020). Technical Report.

[15] Bobbink R., Braun S., Nordin A., Power S., Schütz K., Strengbom J., Weijters M., Tomassen H. (2011).

[16] Gelman A., Hill J. (2006).

[17] Gelman A., Goodrich B., Gabry J., Vehtari A. (2019). R-squared for Bayesian Regression Models. Am. Stat..

[18] Sala O.E., Stuart Chapin F., III, Armesto J.J., Berlow E., Bloomfield J., Dirzo R., Huber-Sanwald E., Huenneke L.F., Jackson R.B., Kinzig A., Leemans R., Lodge D.M., Mooney H.A., Oesterheld M., Poff N.L., Sykes M.T., Walker B.H., Walker M., Wall D.H. (2000). Global Biodiversity Scenarios for the Year 2100. Science (80-.)..

[19] Stevens C.J. (2016). How long do ecosystems take to recover from atmospheric nitrogen deposition?. Biol. Conserv..

[20] Bobbink R., Hicks K., Galloway J., Spranger T., Alkemade R., Ashmore M., Bustamante M., Cinderby S., Davidson E., Dentener F., Emmett B., Erisman J.-W., Fenn M., Gilliam F., Nordin A., Pardo L., De Vries W. (2010). Global assessment of nitrogen deposition effects on terrestrial plant diversity: a synthesis. Ecol. Appl..

[21] Stevens C.J., David T.I., Storkey J. (2018). Atmospheric nitrogen deposition in terrestrial ecosystems: Its impact on plant communities and consequences across trophic levels. Funct. Ecol..

[22] IPBES (2019). Technical Report.

[23] de Vries W., Posch M., Sverdrup H.U., Larssen T., de Wit H.A., Bobbink R., Hettelingh J.-P. (2015). Crit. Loads Dyn. Risk Assessments.

[24] J. Nilsson, P. Grennfelt, N.C. of Ministers, Critical loads for sulphur and nitrogen. Report from a workshop held at Skokloster, Sweden, 19-24 March, 1988, 1988.

[25] Du E. (2022). Handb. Air Qual. Clim. Chang..

[26] Braun S., Schindler C., Rihm B. (2017). Growth trends of beech and Norway spruce in Switzerland: The role of nitrogen deposition, ozone, mineral nutrition and climate. Sci. Total Environ..

[27] Groffman P.M., Baron J.S., Blett T., Gold A.J., Goodman I., Gunderson L.H., Levinson B.M., Palmer M.A., Paerl H.W., Peterson G.D., Poff N.L., Rejeski D.W., Reynolds J.F., Turner M.G., Weathers K.C., Wiens J. (2006). Ecological Thresholds: The Key to Successful Environmental Management or an Important Concept with No Practical Application?. Ecosystems.

[28] Beckage B., Joseph L., Belisle P., Wolfson D.B., Platt W.J. (2007). Bayesian change-point analyses in ecology. New Phytol..

