# Supplementary material for: Hierarchical change-point regression models including random effects to estimate empirical critical loads for nitrogen using Bayesian Regression Models (brms) and JAGS
Source: MethodsX. 2022 Oct 30;9:101902. doi: 10.1016/j.mex.2022.101902 (PMC9647564; doi:10.1016/j.mex.2022.101902)
Supplement: Supplementary Data S1 — RMarkdown output as .pdf file with all R codes used for this paper. This is open data under the CC BY license http://creativecommons.org/licenses/by/4.0/ [file mmc1.pdf]

R script for the paper by Roth et al.

Bayesian change-point regression models including random effects to estimate empirical critical loads for nitrogen

Simon Tresch, Institute for Applied Plant Biology (IAP), Witterswil, Switzerland
Tobias Roth, University of Basel, Zoological Institute and Hintermann & Weber, Reinach, Switzerland

19 September 2022

Contents

1 Used packages **2**

2 Load data **2**
2.1 Data management 3

3 Data inspection **4**

4 Estimating CLempN with Bayesian change-point regression using JAGS (BCR_JAGS) **6**
4.1 Plotting CLempN predictions 8

5 Estimating CLempN with Bayesian change-point regression using brms (BCR_brms) **11**
5.1 Plotting CLempN predictions 14

References **16**

Important notes This RMarkdown was created using the R version 4.0.3 (2020-10-10) (R Core Team and Team R Development Core 2021).

This scripts includes data preparation and all statistical models including model diagnostics and plots used for the paper.

All codes are coded by the main authors, based on literature given in the main paper and supplementary material.

In case of any questions please contact the main author.

Please cite the main paper Roth et al. 2022 MethodsX

1 Used packages

```
library(tidyverse)
```

Used for data manipulation (dplyr) and plotting (ggplot) see Wickham et al. (2019).

```
library(ggeffects)
```

Marginal effects and estimated marginal means from regression models see Lüdecke (2018).

```
library(ggpubr) # multiple ggplots
library(jtools) # model summary outputs
library(readxl) # import xlsx data
library(xtable) # print LaTex tables
```

```
library(lme4) # LMEM and GLMEM
```

Mixed effect models Bates et al. (2015).

```
library(blmeco) # Bayesian Data Analysis
```

Bayesian Data Analysis see Korner-Nievergelt et al. (2015).

```
library(rjags) # MCMC simulations
```

MCMC simulations of the change-point models were conducted using JAGS, version 4.3.0 Plummer (2003), executed in R using rjags Plummer (2019).

```
library(brms) # brm::brms
```

Bayesian Multilevel Models Using Stan see Bürkner (2017).

2 Load data

Data provided by Du, Doorn, and Vries (2021). Compare with Fig. 3: Relative importance of the seven potential variables affecting the spatial variation of leaf N:P ratio and the conditional regression plots for the most important drivers in Du, Doorn, and Vries (2021).

```
mydata<- as_tibble(read.csv("data/du_et_al_2021_data_fig3.csv",header = TRUE,
                  sep=",",dec = "."))
mydata<-mydata %>% mutate(Species=as.factor(Species))

mydata<- mydata %>% dplyr::filter(Forest_category=="Conifer") %>% droplevels()
#10 conifer species

mydata<-mydata %>% tidyr::separate(plot_country, c("plot", "country"))  %>% mutate(plot=as.factor(as.cha


mydata
```

```
## # A tibble: 103 x 14
##    plot  country   lat   lon code_tree_species Species   Npratio Forest_category
##    <fct> <fct>   <dbl> <dbl>             <int> <fct>       <dbl> <chr>
##  1 69    1        42.2  8.83               129 Pinus ni~   11.1  Conifer
##  2 71    1        41.8  9.2                130 Pinus pi~   10.7  Conifer
##  3 76    1        44.0  6.67               134 Pinus sy~    9.73 Conifer
##  4 68    1        44.7  6.55               116 Larix de~    9.06 Conifer
##  5 90    1        44.5  6.45               100 Abies al~    9.49 Conifer
```

```
##  6 43    1        45.6  6.78              118 Picea ab~    7.45 Conifer
##  7 96    1        45.4  6.12              100 Abies al~   11.0  Conifer
##  8 25    11       38.5 -0.62              125 Pinus ha~   13.0  Conifer
##  9 84    1        48.8  7.7               134 Pinus sy~   13.0  Conifer
## 10 13    14       46.6 15.5               118 Picea ab~    9.40 Conifer
## # ... with 93 more rows, and 6 more variables: MAT <dbl>, MAP <dbl>,
## #   Clay <int>, Soil_CNrt <int>, Ndep <dbl>, Age <dbl>
```

2.1 Data management

```
# new tree species (n=5) based on genus types
mydata <- mydata %>% mutate(tree_species = dplyr::if_else(Species %in% c("Pinus halepensis",
                    "Pinus nigra","Pinus pinaster","Pinus pinea","Pinus sylvestris" ),
                    "Pinus (n=5)", as.character(Species))) %>%
  mutate(tree_species = dplyr::if_else(tree_species %in% c("Picea abies", "Picea sichensis"),
                                "Picea (n=2)", as.character(tree_species)))

levels(as.factor(mydata$tree_species))
```

```
## [1] "Abies alba"            "Larix decidua"         "Picea (n=2)"
## [4] "Pinus (n=5)"           "Pseudotsuga menziesii"
```

3 Data inspection

```
hist(mydata$Npratio)
```

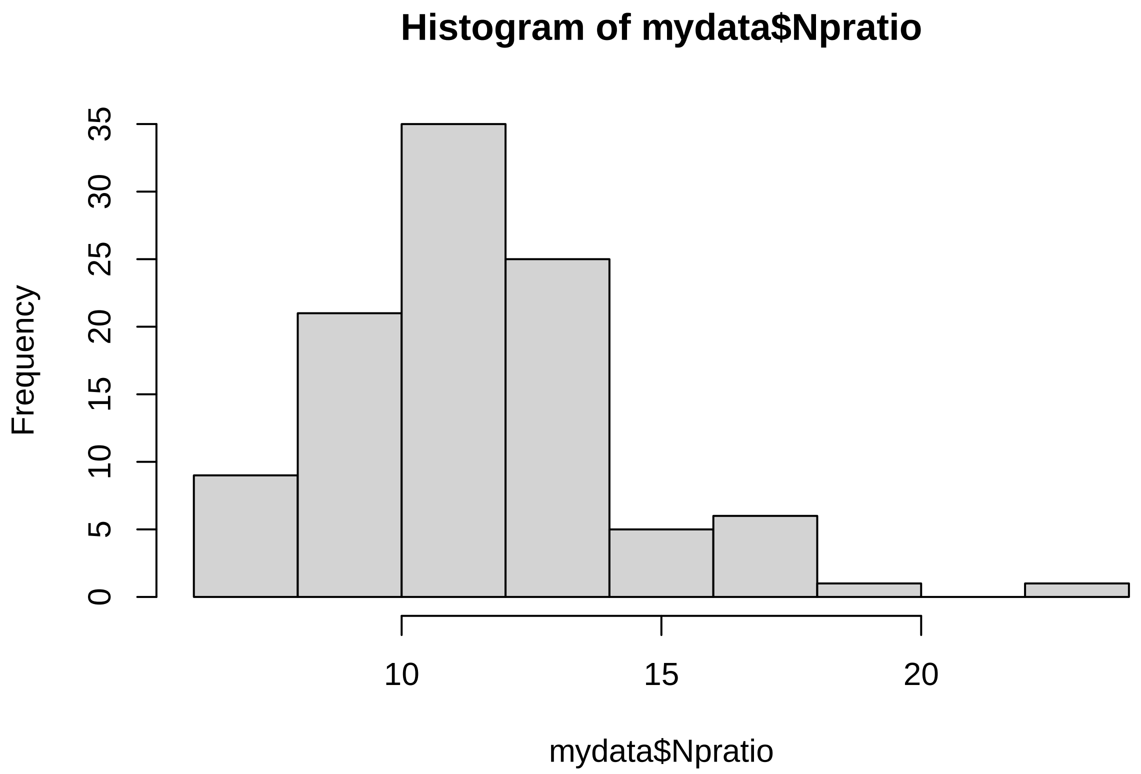

Most likely Poisson distribution, which is a discrete probability distribution that naturally describes the distribution of count data Korner-Nievergelt et al. (2015).

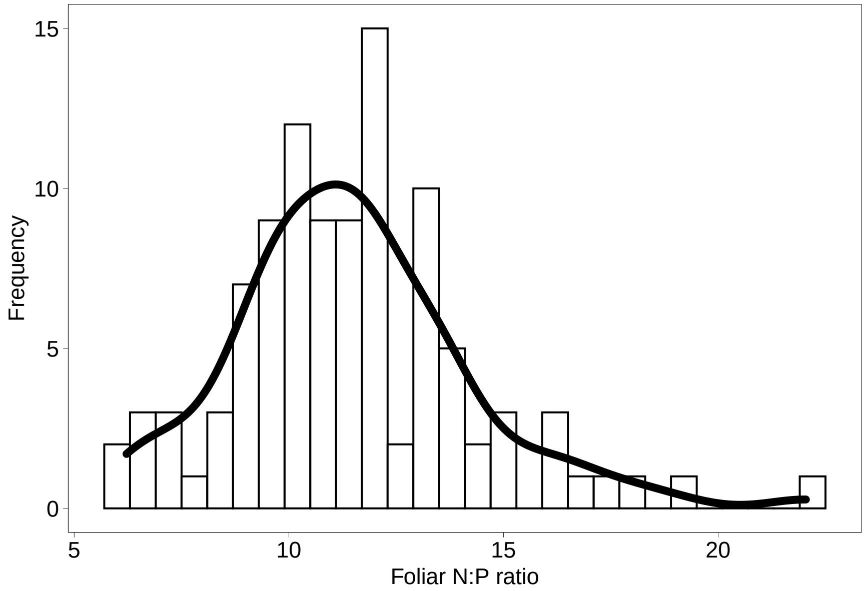

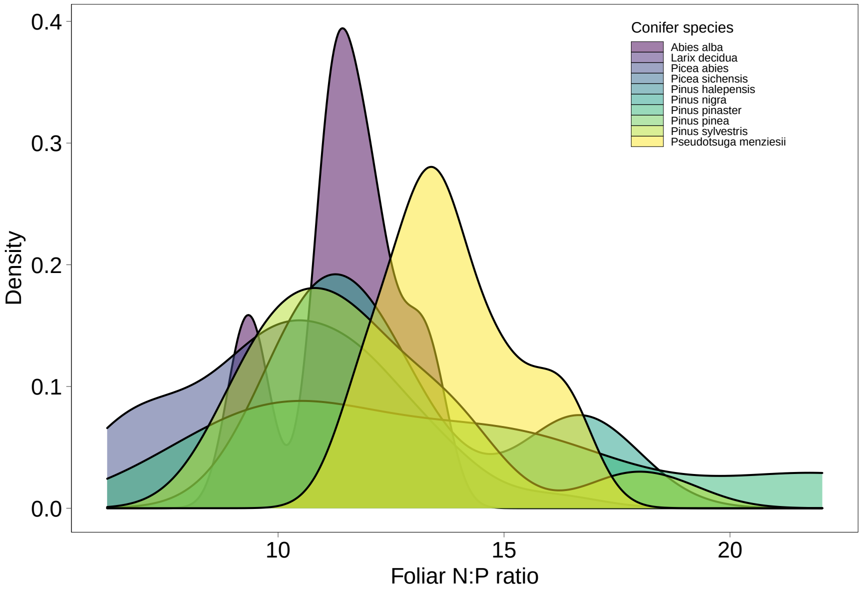

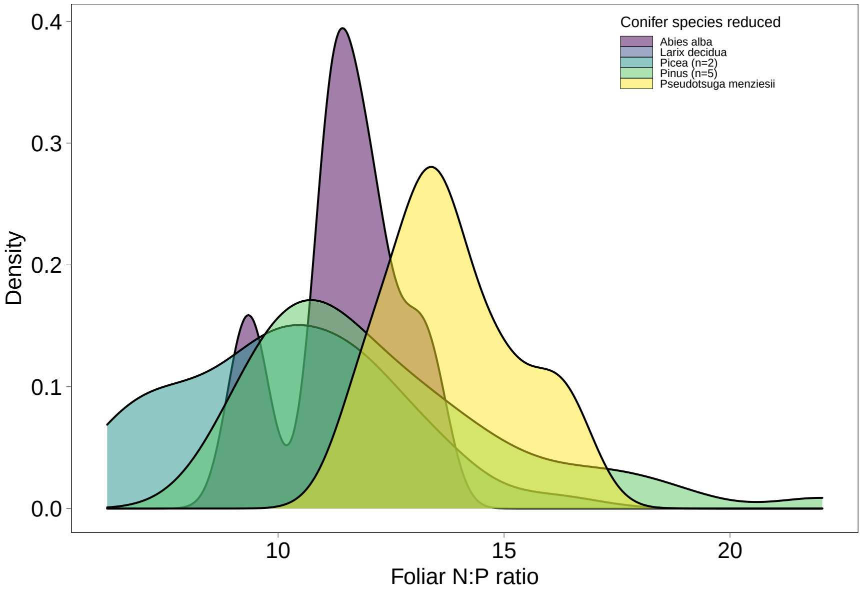

Tree species might have an influence on outcome variable foliar N:P ratio according to histogram plots. Thus, the estimation of a CLempN reflecting hierarchical data structure need to take into account the random effect of tree species.

4 Estimating CLempN with Bayesian change-point regression using JAGS (BCR_JAGS)

Adapted change-point regression approach using JAGS.

```
# JAGS Settings
t.n.thin <-      2
t.n.chains <-    2
t.n.burnin <- 50000
t.n.iter <-   100000

# Function to create initial values
inits <- function() {
  list(
    CLempN = rnorm(1, 10, 5),
    beta1 = rnorm(1, 0, 0.1),
    betaN = rnorm(1, 0, 0.1),
    mu = runif(1, -5, 5),
    musd = runif(1, 0, 5)
  )
}
```

We used the approved critical loads according to Bobbink et al. (2011) to construct an informative prior for the critical loads in the gradient studies. We assumed a normal distribution with the approved critical load as its mean and half the range as its standard deviation -> Normal(mean = 15, sd = 5).

```
# Prepare data for JAGS
ntree_species <- mydata %>% dplyr::select(tree_species) %>% unique() %>% count()
ntree_species <- as.integer(ntree_species)#number of random effects now 5 tree species
tree_species<- as.integer(factor(mydata$tree_species))
obs = mydata %>% nrow()
N_P <- mydata$Npratio
N <- mydata$Ndep# Time series data on total N deposition were based on
# results of the EMEP MSC-W model (0.1° resolution) (EMEP, 2019).
N_s = scale(N)
MAT_s = mydata$MAT-10#mean annual temperature Time series data (1995-2017) on MAT and MAP
# for each plot were retrieved from the Europe-wide E-OBS ensemble climate dataset
# (0.25° resolution)


### mod with random effect ####
# Prepare data for JAGS
datjags <- list(dependvar = N_P,
                random=tree_species,
                nrandom=ntree_species,
                obs=obs,
                fixeff = MAT_s,
                N=N)
# Run Jags
jagres <- jags.model('JAGS/JAGS_change_point_model_random_effect.R',data = datjags,
                     n.chains = t.n.chains, inits = inits, n.adapt = t.n.burnin)
```

```
## Compiling model graph
##    Resolving undeclared variables
##    Allocating nodes
```

```
## Graph information:
##    Observed stochastic nodes: 103
##    Unobserved stochastic nodes: 11
##    Total graph size: 927
##
## Initializing model
```

```
params <- c("betaN", "beta1", "CLempN")

BCR_JAGS <- coda.samples(jagres, params, n.iter=t.n.iter, thin=t.n.thin)

# convergence diagnostics with coda
par(mfrow=c(2,3), mar=c(4,4,2,1), mgp=c(2.2,0.8,0))
plot(BCR_JAGS) #Left: trace plot->it shows the values the parameter took during the runtime
```

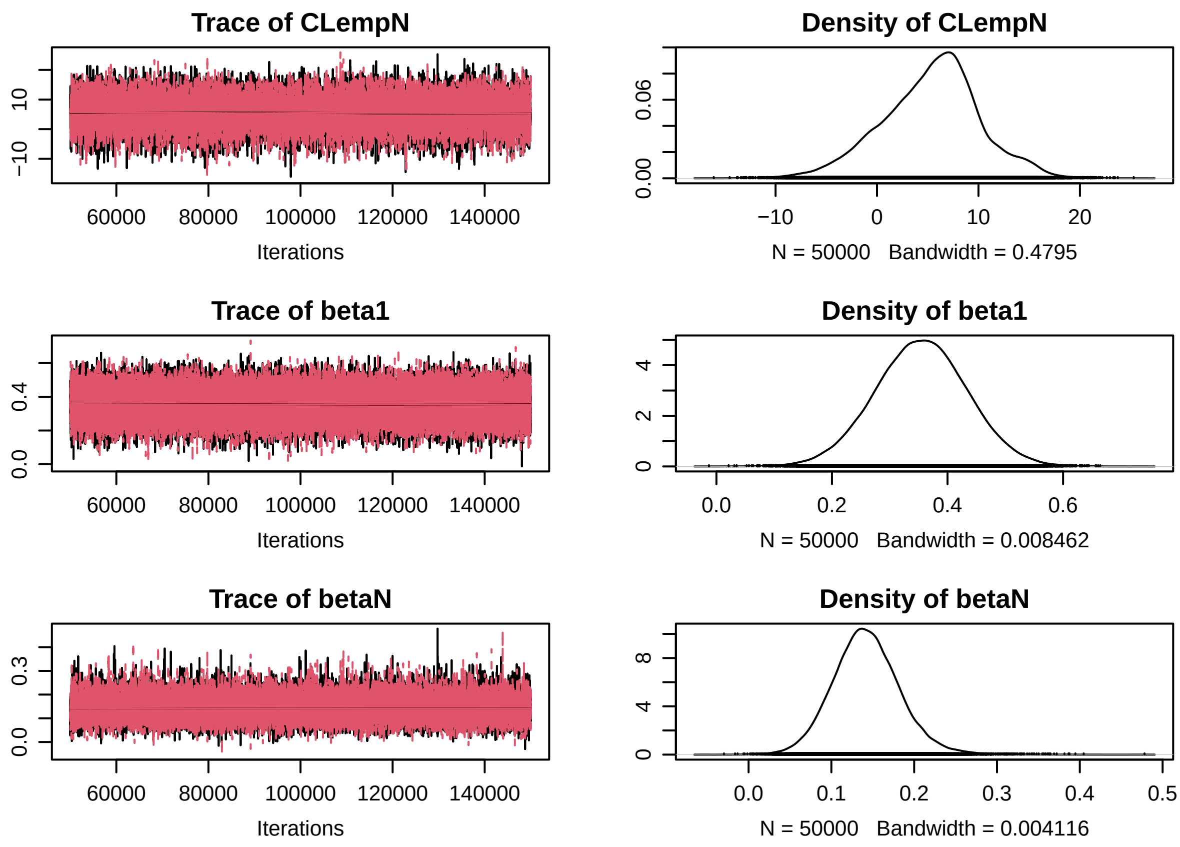


```
# of the chain.
# Right: marginal density plot->the distribution of the values of the parameter
# in the chain.

# scale reduction factors by Gelman-Rubin
# measures whether there is a significant difference between the variance within several
# chains and the variance between several chains by a value ->"scale reduction factors"
gelman.diag(BCR_JAGS)
```

```
## Potential scale reduction factors:
##
##        Point est. Upper C.I.
```

```
## CLempN          1          1
## beta1           1          1
## betaN           1          1
##
## Multivariate psrf
##
## 1
```

```
# scale reduction factors of 1 means that between variance and within chain variance
# are equal, # larger values mean that there is still a notable difference between chains
# as a rule of thumb < 1.1  -> OK

CL<- as.data.frame(summary(BCR_JAGS)$quantiles)
CL<- do.call(rbind.data.frame, BCR_JAGS)
CLempN_table<-as.data.frame(round(median(CL$CL),2))#median
colnames(CLempN_table)<-c("BCR_JAGS_median")
CLempN_table$BCR_JAGS_sd<-sd(CL$CL) #SD
rownames(CLempN_table)<-c("BCR_JAGS")

# CLempN estimation with BCR_JAGS:
CLempN_table
```

```
##          BCR_JAGS_median BCR_JAGS_sd
## BCR_JAGS            5.71    4.812788
```

4.1 Plotting CLempN predictions

```
# range of N deposition based on gradient study
ndep<- (min(mydata$Ndep):max(mydata$Ndep))


mod_sum_median<-summary(BCR_JAGS)$quantiles[,"50%"]
mod_sum_median
```

```
##    CLempN     beta1     betaN
## 5.7060101 0.3547331 0.1408969
```

```
#Mean foliar N:P ratio
median<-median(mydata$Npratio)
median
```

```
## [1] 11.20623
```

```
pred_median<-median+(ndep>mod_sum_median["CLempN"]) * mod_sum_median["betaN"] * (ndep-mod_sum_median["C
pred_median
```

```
##  [1] 11.20623 11.20623 11.20623 11.20623 11.20623 11.22744 11.36834 11.50924
##  [9] 11.65013 11.79103 11.93193 12.07282 12.21372 12.35462 12.49551 12.63641
## [17] 12.77731 12.91820 13.05910 13.20000 13.34090 13.48179 13.62269 13.76359
## [25] 13.90448 14.04538 14.18628 14.32717 14.46807 14.60897 14.74986 14.89076
## [33] 15.03166 15.17256 15.31345 15.45435 15.59525 15.73614
```

```r
plot(ndep,pred_median,ty="l")
```

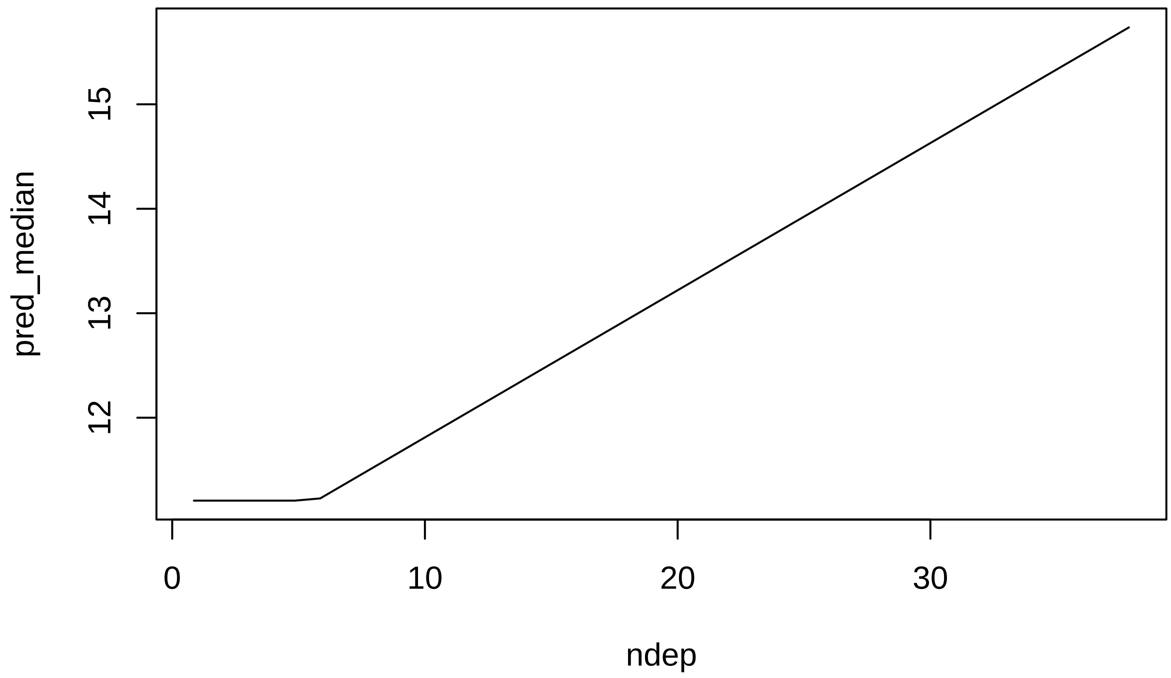


```r
pred_data<-bind_cols(pred_median,ndep)
colnames(pred_data)<-c("pred_median","ndep")

# CrI

post_samples<-rbind(BCR_JAGS[[1]][seq(1, t.n.iter/t.n.thin,t.n.thin),],# chain 1
                    BCR_JAGS[[2]][seq(1, t.n.iter/t.n.thin,t.n.thin),])# chain 2
dim(post_samples)
```

```
## [1] 50000     3
```

```r
pred_CrI<-array(NA,dim = c(nrow(post_samples),length(ndep)))

for(i in 1: nrow(post_samples)) {
  pred_CrI[i,]<-median+(ndep>post_samples[i,"CLempN"]) *post_samples[i,"betaN"] * (ndep-post_samples[i,
}




pred_data$lower_CrI<-apply(pred_CrI,2,quantile,probs=0.025)
pred_data$upper_CrI<-apply(pred_CrI,2,quantile,probs=0.975)
pred_data
```

```
## # A tibble: 38 x 4
```

```
##    pred_median  ndep lower_CrI upper_CrI
##          <dbl> <dbl>     <dbl>     <dbl>
##  1        11.2 0.857      11.2      12.0
##  2        11.2 1.86       11.2      12.2
##  3        11.2 2.86       11.2      12.3
##  4        11.2 3.86       11.2      12.5
##  5        11.2 4.86       11.2      12.7
##  6        11.2 5.86       11.2      12.8
##  7        11.4 6.86       11.2      13.0
##  8        11.5 7.86       11.2      13.2
##  9        11.7 8.86       11.2      13.3
## 10        11.8 9.86       11.2      13.5
## # ... with 28 more rows
```

```
# CL from change point model
sq <- summary(BCR_JAGS)$quantiles
(lower_CL<-sq["CLempN","2.5%"])
```

```
## [1] -4.696867
```

```
(upper_CL<-sq["CLempN","97.5%"])
```

```
## [1] 14.81338
```

```
(mean_CL<-sq["CLempN","50%"])
```

```
## [1] 5.70601
```

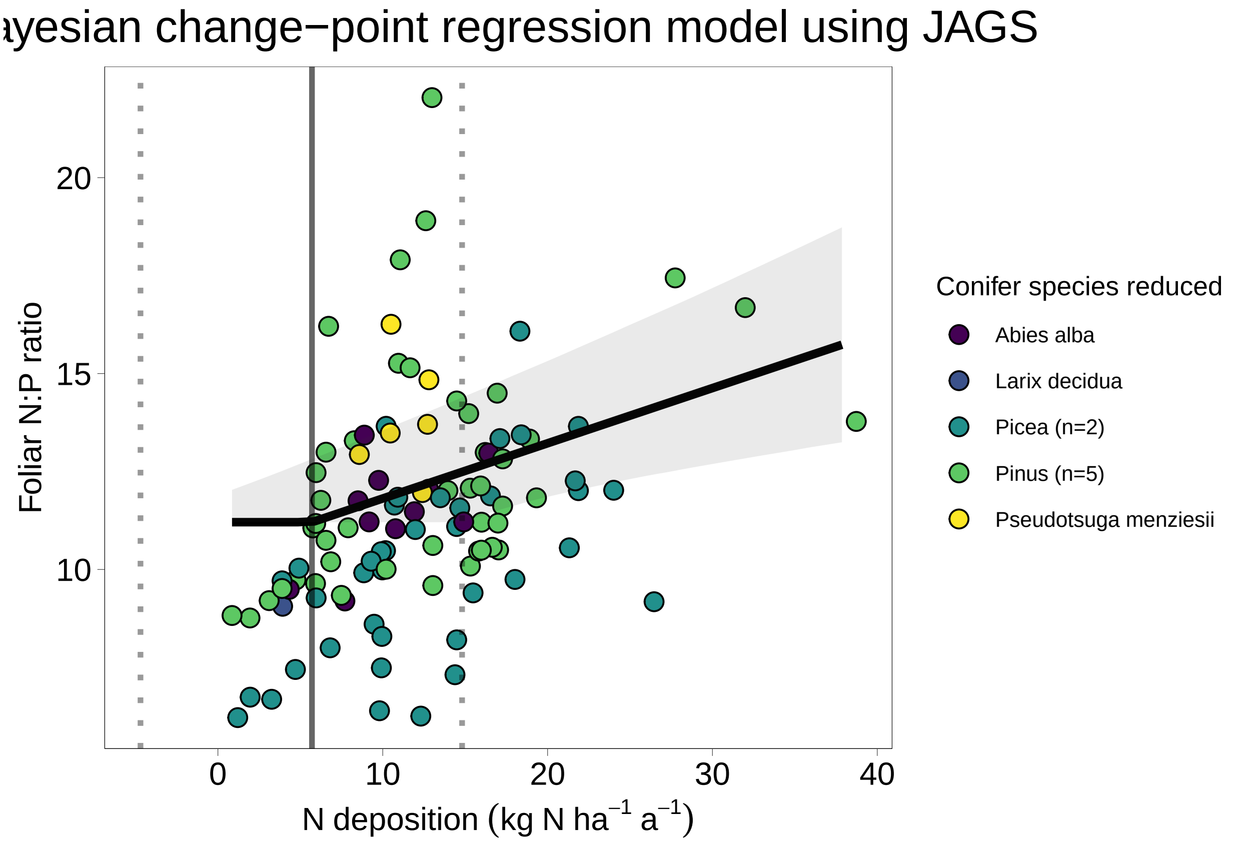

5 Estimating CLempN with Bayesian change-point regression using brms (BCR_brms)

Adapted change-point regression approach using brms Bürkner (2017).

We used the approved critical loads according to Bobbink et al. (2011) to construct an informative prior for the critical loads in the gradient studies. We assumed a normal distribution with the approved critical load as its mean and half the range as its standard deviation -> Normal(mean = 15, sd = 5).

```
# Prepare data for brms

mydata$dependvar <- mydata$Npratio
mydata$fixeff <- mydata$MAT-10#mean annual temperature Time series data (1995-2017) on MAT and MAP
mydata$Ndep <- mydata$Ndep# Time series data on total N deposition were based on results
# of the EMEP MSC-W model (0.1° resolution) (EMEP, 2019).
mydata$random<- as.integer(factor(mydata$Species)) #random effect tree species


# Priors ####
bprior <-
  prior(normal(10, 5), nlpar = "beta0") +
  prior(normal(0, 2),  nlpar = "beta1") +
  prior(normal(0, 2),  nlpar = "betaN") +
  prior(normal(10, 5), nlpar = "CLempN")


# mod random intercepts and CLempN ####

changepoint_model <- brmsformula(
  dependvar ~ beta0 + beta1 * fixeff +
    step(Ndep - CLempN) * betaN * (Ndep - CLempN),
  CLempN ~ 1+ (1|random), #group level effect of tree species for CLempN
  beta0 ~ 1+ (1|random), #group level effect of tree species on intercepts
  beta1 + betaN  ~ 1,
  nl = T,
  family = gaussian
)

# Model fit with brms::brm using Stan ####
CPR_brms <-
  brms::brm(changepoint_model,
                          family = student(link = "identity"),
                          data = mydata,
                          prior = bprior,
                          chains = 2,
                          cores = 4,
                          warmup = 50000,
                          thin = 2,
                          iter=100000)


print(CPR_brms, digits = 1, robust = TRUE)
```

```
##  Family: gaussian
##   Links: mu = identity; sigma = identity
```

```
## Formula: dependvar ~ beta0 + beta1 * fixeff + step(Ndep - CLempN) * betaN * (Ndep - CLempN)
##          CLempN ~ 1 + (1 | random)
##          beta0 ~ 1 + (1 | random)
##          beta1 ~ 1
##          betaN ~ 1
##    Data: mydata (Number of observations: 103)
##   Draws: 2 chains, each with iter = 1e+05; warmup = 50000; thin = 2;
##          total post-warmup draws = 50000
##
## Group-Level Effects:
## ~random (Number of levels: 10)
##                      Estimate Est.Error l-95% CI u-95% CI Rhat Bulk_ESS
## sd(CLempN_Intercept)      2.0       1.7      0.1      7.6  1.0    38647
## sd(beta0_Intercept)       0.5       0.4      0.0      1.7  1.0    27590
##                      Tail_ESS
## sd(CLempN_Intercept)    36971
## sd(beta0_Intercept)     34288
##
## Population-Level Effects:
##                  Estimate Est.Error l-95% CI u-95% CI Rhat Bulk_ESS Tail_ESS
## CLempN_Intercept      9.1       4.1      0.5     17.6  1.0    39592    41052
## beta0_Intercept      11.4       0.5     10.3     12.3  1.0    38627    37547
## beta1_Intercept       0.4       0.1      0.3      0.6  1.0    40278    41562
## betaN_Intercept       0.1       0.1      0.0      0.3  1.0    40393    37362
##
## Family Specific Parameters:
##       Estimate Est.Error l-95% CI u-95% CI Rhat Bulk_ESS Tail_ESS
## sigma      2.1       0.2      1.8      2.4  1.0    43549    42831
##
## Draws were sampled using sampling(NUTS). For each parameter, Bulk_ESS
## and Tail_ESS are effective sample size measures, and Rhat is the potential
## scale reduction factor on split chains (at convergence, Rhat = 1).
```

```
bayes_R2(CPR_brms) #R-squared for Bayesian regression models
```

```
##     Estimate  Est.Error      Q2.5     Q97.5
## R2 0.4385973 0.05749342 0.3136496 0.5374953
```

```r
# Model convergence diagnostics
par(mfrow=c(2,7), mar=c(4,4,2,1), mgp=c(2.2,0.8,0))
plot(CPR_brms)
```

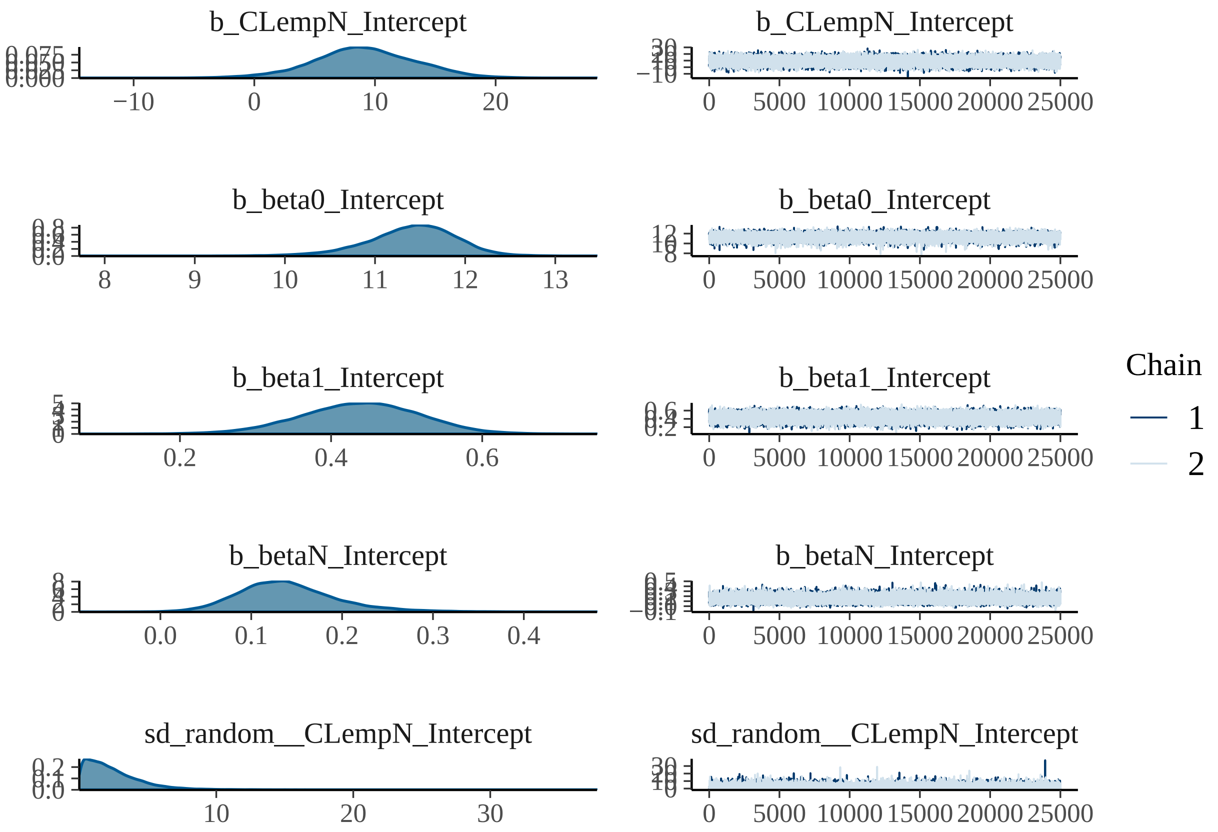

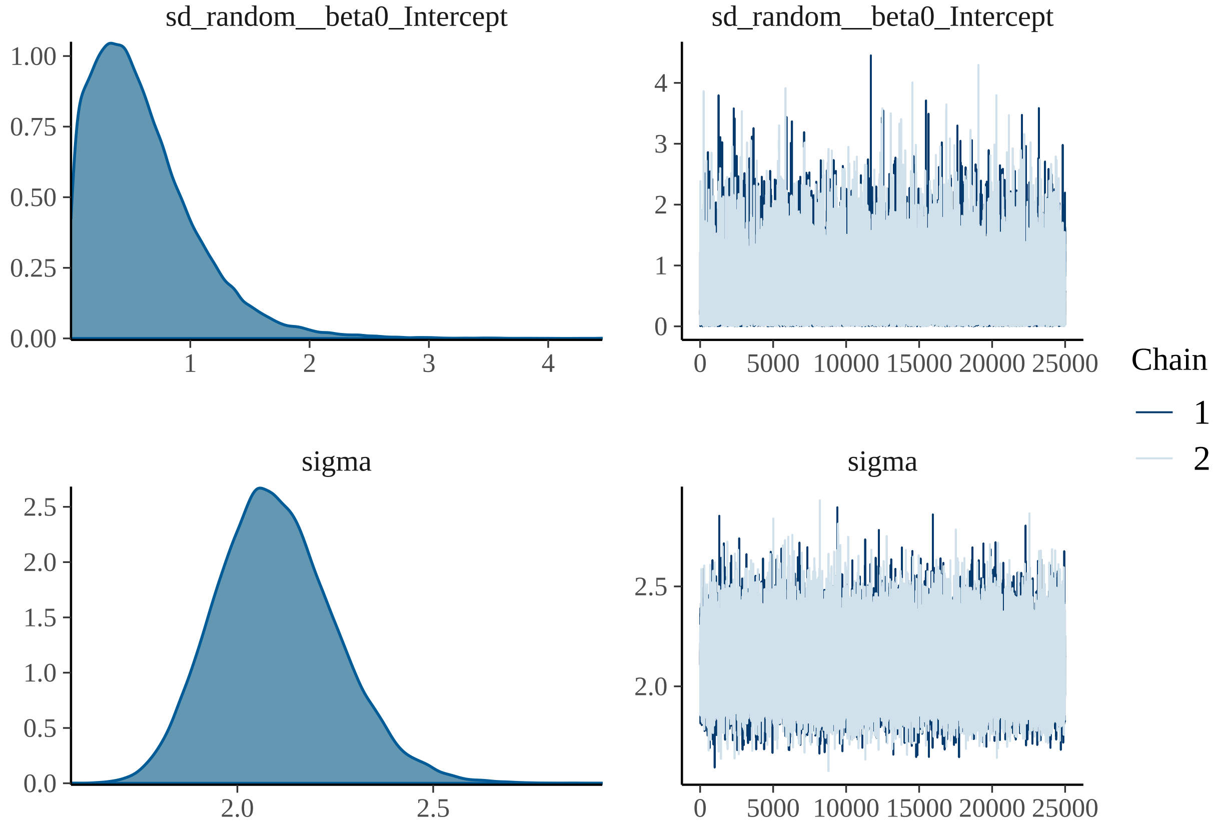


```
CL<- as.data.frame(CPR_brms)
CLempN_table<-as.data.frame(round(median(CL$b_CLempN_Intercept),2))#median
colnames(CLempN_table)<-c("CPR_brms_median")
CLempN_table$CPR_brms_sd<-round(sd(CL$b_CLempN_Intercept),2) #SD
rownames(CLempN_table)<-c("CPR_brms")

# CLempN estimation with BCR_brms:
CLempN_table
```

```
##          CPR_brms_median CPR_brms_sd
## CPR_brms            9.06        4.31
```

5.1 Plotting CLempN predictions

```
## # Predicted values of dependvar
##
##  Ndep | Predicted |         95% CI
## -------------------------------------
##  0.86 |     10.96 | [ 9.92, 11.81]
##  5.76 |     10.99 | [10.10, 11.82]
##  7.90 |     11.06 | [10.23, 11.86]
##  9.92 |     11.18 | [10.36, 11.98]
## 11.66 |     11.32 | [10.50, 12.14]
## 13.95 |     11.56 | [10.69, 12.46]
## 15.94 |     11.81 | [10.89, 12.77]
## 38.73 |     14.91 | [12.51, 17.90]
##
```

```
## Adjusted for:
## * fixeff = -1.09
```

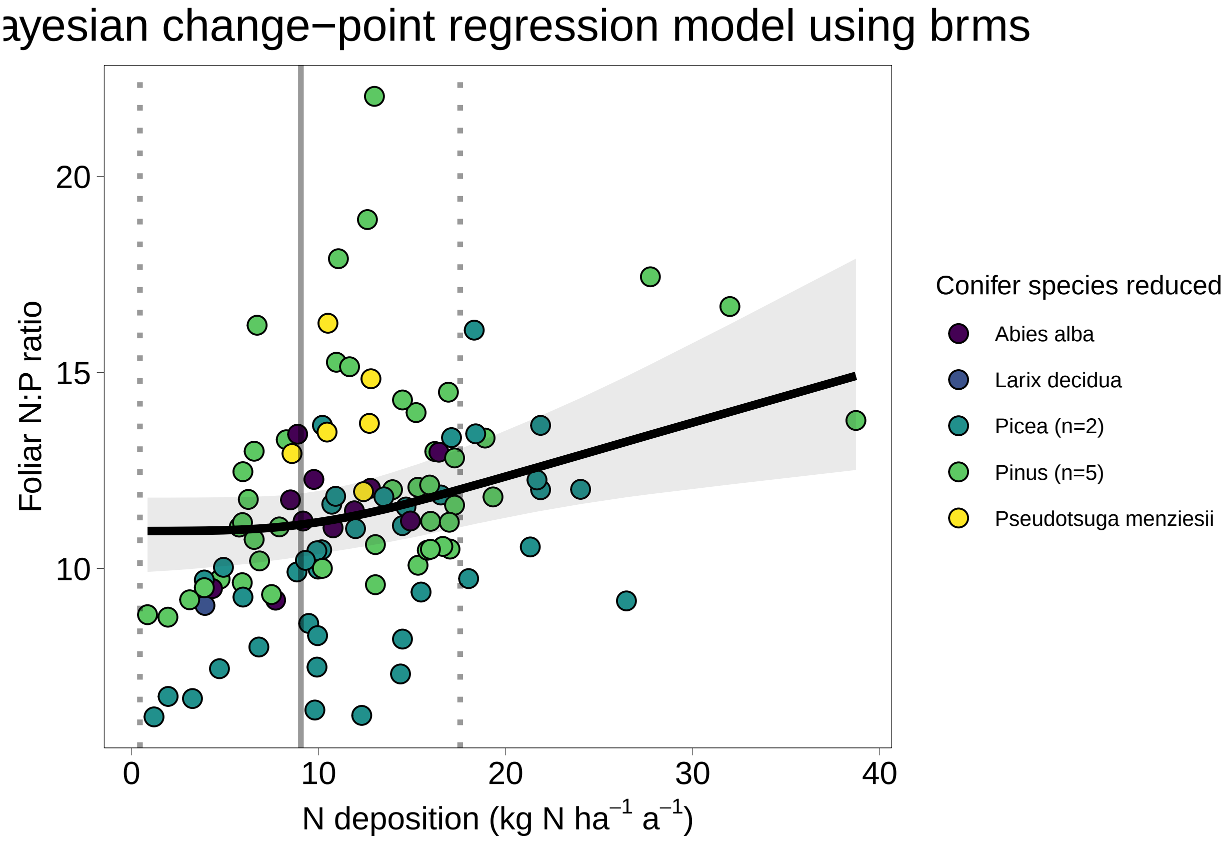

References

Bates, D, M Maechler, B Bolker, and S Walker. 2015. "Fitting Linear Mixed-Effects Models Using lme4." *J. Stat. Softw.* 67 (1): 1–48.

Bobbink, Roland, Sabine Braun, Annika Nordin, Sally Power, Kirsten Schütz, Joachim Strengbom, Maaike Weijters, and Hilde Tomassen. 2011. *Review and revision of empirical critical loads and dose-response relationships. Proceedings of an expert workshop, Noordwijkerhout, 23-25 June 2010.* CCE. http://www.rivm.nl/bibliotheek/rapporten/680359002.pdf.

Bürkner, Paul-Christian. 2017. "brms : An R Package for Bayesian Multilevel Models Using Stan." *J. Stat. Softw.* 80 (1). https://doi.org/10.18637/jss.v080.i01.

Du, Enzai, Maarten van Doorn, and Wim de Vries. 2021. "Spatially divergent trends of nitrogen versus phosphorus limitation across European forests." *Sci. Total Environ.* 771 (June): 145391. https://doi.org/10.1016/j.scitotenv.2021.145391.

Korner-Nievergelt, Franzi, Tobias Roth, Stefanie Von Felten, Jérôme Guélat, Bettina Almasi, and Pius Korner-Nievergelt. 2015. *Bayesian Data Analysis in Ecology Using Linear Models with R, BUGS, and STAN.* Elsevier. https://doi.org/10.1016/C2013-0-23227-X.

Lüdecke, Daniel. 2018. "ggeffects: Tidy Data Frames of Marginal Effects from Regression Models." *J. Open Source Softw.* 3 (26): 772. https://doi.org/10.21105/joss.00772.

Plummer, Martyn. 2003. "JAGS: A Program for Analysis of Bayesian Graphical Models using Gibbs Sampling." *3rd Int. Work. Distrib. Stat. Comput. (DSC 2003); Vienna, Austria* 124.

———. 2019. *rjags: Bayesian Graphical Models using MCMC.* https://cran.r-project.org/package=rjags.

R Core Team, and Team R Development Core. 2021. "R: A Language and Environment for Statistical Computing." Vienna, Austria: R Foundation for Statistical Computing; R Foundation for Statistical Computing. http://www.r-project.org/.

Wickham, Hadley, Mara Averick, Jennifer Bryan, Winston Chang, Lucy McGowan, Romain François, Garrett Grolemund, et al. 2019. "Welcome to the Tidyverse." *J. Open Source Softw.* 4 (43): 1686. https://doi.org/10.21105/joss.01686.
